# CRISPR activation of *DLX5* drives neural progenitors to the GnRH cell fate

**DOI:** 10.1530/JME-26-0040

**Published:** 2026-07-23

**Authors:** Shrinidhi Madhusudan, Nazli Eskici, Celia Gomez-Sanchez, Kristiina Pulli, Kirsi Vaaralahti, Venkatram Yellapragada, James R W Conway, Yafei Wang, Taneli Raivio

**Affiliations:** ^1^ Stem Cells and Metabolism Research Program (STEMM), Research Programs Unit, Faculty of Medicine, University of Helsinki, Helsinki, Finland; ^2^ Medicum, Faculty of Medicine, University of Helsinki, Helsinki, Finland; ^3^ Estonian Genome Center, Institute of Genomics, University of Tartu, Tartu, Estonia; ^4^ Turku Bioscience Centre, University of Turku and Åbo Akademi University, Turku, Finland; ^5^ Department of Biochemistry and Developmental Biology, Faculty of Medicine, University of Helsinki, Helsinki, Finland; ^6^ Pediatric Research Center, Helsinki University Hospital, New Children’s Hospital, Helsinki, Finland

**Keywords:** GnRH neurons, *DLX5*, motility, CRISPR

## Abstract

Gonadotropin-releasing hormone (GnRH) neurons regulate the hypothalamic–pituitary–gonadal (HPG) axis and are required for puberty onset and reproductive competence. However, the transcriptional regulators governing GnRH neuron specification and migration remain poorly defined. The homeodomain transcription factor *DLX5* is expressed in fetal human GnRH neurons, its expression precedes that of *GNRH1* in human pluripotent stem cell (hPSC)-derived GnRH neurons, and in mice, it serves as a guidance cue for GnRH neuron migration. We hypothesized that *DLX5* may act as an upstream regulator of human GnRH neuron fate specification and migratory capacity. Using CRISPR activation, we upregulated *DLX5* during FGF8b-directed differentiation of hPSCs to GnRH neurons via dual SMAD inhibition and Notch inhibition, as previously described. *DLX5* activation increased neural progenitor motility (*P* < 0.001), upregulated *FGF8* (*P* < 0.05), and induced GABAergic markers, including *GAD1* and *GAD2*. Notably, *DLX5* activation induced *GNRH1* in the absence of exogenous FGF8b (*P* < 0.05), suggesting that in GnRH neurons, *DLX5* regulates *FGF8*. When combined with exogenous FGF8b, *DLX5* activation produced distinct neuronal patterning accompanied by upregulation of extracellular matrix genes, such as *SPARC*, which has been implicated in neurite outgrowth. Collectively, these data indicate that activation of *DLX5* promotes GnRH neurogenesis from hPSCs, by driving GABAergic fate, inducing *FGF8*, and remodeling the extracellular matrix.

## Introduction

GnRH neurons control puberty and fertility by stimulating the release of luteinizing hormone and follicle-stimulating hormone from the pituitary (1). GnRH neurons originate in the olfactory placodes (OPs) and migrate along olfactory sensory neurons (OSNs) (2, 3, 4) and terminal nerve axons (3, 4, 5) into the hypothalamus in the forebrain (6). GnRH neurons exhibit an autoregulatory capacity, modulating their own migration and axon guidance (7). Aberrations in GnRH neuron development, migration, and/or secretion of GnRH decapeptide can lead to variations in puberty timing and a wide range of reproductive disorders (4, 6).

There are six distal-less homeobox transcription factors (*DLX1–DLX6*) that are highly conserved across all vertebrates and regulate several developmental pathways both within and outside the brain (8). Homeobox genes regulate body patterning (9), craniofacial development (10), forebrain development (8, 11, 12), and neuron migration (9, 13). The six *Dlx* genes are expressed in bigenic clusters (14, 15), where *Dlx1/2* and *Dlx5/6* are expressed in the forebrain (8). *Dlx5/6* is expressed in both postmitotic differentiating neurons (16) and hypothalamic GABAergic neurons (8, 17). The transcription factors *Dlx5*, *Dlx3*, and *Pax6* serve as established markers of OP precursors (18), and one of the earliest recorded expressions of *Dlx5* was shown in the mouse at 6.75 dpc right before the formation of the neural plate, where it regulated the formation of the anterior neural ridge (19). *Dlx5* is expressed around the OPs in ring-like structures at E8.5–9 (20) and enables growth cone properties of the olfactory receptor neurons to form appropriate connections to the forebrain (21). *Dlx5* is also expressed in the olfactory bulbs (OBs) and the olfactory epithelium (OE) and plays an important role in the development of these systems (22, 23). DLX factors have been shown to bind ATTA homeodomain motifs near the *Gnrh1* promoter both *in vitro* and *in vivo*, thereby directly regulating *Gnrh1* transcription (7, 24). However, the role of *DLX5* in GnRH neurogenesis in humans has not been elucidated.

Using our previously published differentiation protocol that sequentially applies dual SMAD inhibition, FGF8b-mediated anterior forebrain patterning, and DAPT-driven Notch inhibition (25, 26, 27), we have demonstrated the generation of GABAergic GnRH neurons from hPSCs. Single-cell transcriptomics revealed that *DLX5* expression precedes *GNRH1* onset, and human fetal tissue confirmed *DLX5* colocalization with migratory GnRH neurons (27). Therefore, we hypothesized that *DLX5* may play a role in human GnRH neuron ontogenesis.

To investigate the role of *DLX5* in GnRH neuron development, we generated a CRISPR activation line in our GnRH Td-Tomato (GnRH-TdT) reporter line (26). Our findings reveal that *DLX5* activation enhances neural progenitor motility and increases *GNRH1* expression. *DLX5* activation also upregulates genes associated with axon guidance, GABAergic identity, and extracellular matrix organization, suggesting a role for *DLX5* in neurogenesis.

## Materials and methods

### Cell culture and GnRH differentiation

Human embryonic stem cells from WiCell (WA09) were used in this study. We used our previously published GnRH-TdT reporter line H9C11 (26), which was single-cell sorted by FACS to create the clonal line D7. Stem cells were cultured in mTESR1 (STEMCELL Technologies, Germany, 85850) monolayer on Matrigel-coated (Corning, Netherlands, Cat. 356231) plasticware (ThermoFisher Scientific, Finland, Cat. 153066) and passaged using 0.5 mM EDTA (ThermoFisher Scientific, Cat. 15575-038) in PBS (Corning, Cat. 21-040-CV). Cells were passaged at a 1:4 ratio, and once 85–90% confluency was achieved, differentiation to *GNRH1* neurons was started. In brief, confluent cells were first treated with 2 μM dorsomorphin (Selleckchem, Germany, Cat. S7306) and 10 μM SB431542 (Sigma, Germany, Cat. S4317) in basal N2B27 (25) medium for 10 days. The medium was refreshed daily. On day 10, cells were split using 200 U/mL collagenase IV (ThermoFisher Scientific, Cat. 17104019) and 10 μM Y-27632 (Selleckchem, Cat. S1049) in a 1:2 ratio and plated on Matrigel-coated dishes using basal N2B27 medium. From days 11 to 20, the cells were treated with 100 ng/mL FGF8b (PeproTech, Finland Cat. AF-100-25) in basal medium refreshed daily. On day 20, the cells were split 1:8 with EDTA and neural progenitor cells (NPCs) were plated on Matrigel-coated dishes. Finally, from days 21 to 25, NPCs were treated with 20 μM of the Notch signaling inhibitor DAPT (Selleckchem, Cat. S2215) and the medium was refreshed every other day. Neurons were collected in cold PBS for downstream analysis.

### CRISPR activation line and guide RNA cloning

We used the previously published CRISPR DDdCas9VP192 system to generate the *DLX5* CRISPR activation line (28). In brief, 12 VP16 domains are fused to the dCas9 complex, paired with a Tet-ON system and dihydrofolate reductase (DHFR)-derived destabilization domain (DD). This inducible system requires both doxycycline (DOXY) and trimethoprim (TMP) to stabilize the protein complex and induce gene activation. We used 2 μg/mL of DOXY (Fisher Scientific, Finland, Cat. BP26531) and 1 μM of TMP (Sigma, Cat. 92131) in basal N2B27 medium, hereby referred to as ‘activation medium’.

Plasmids containing the CRISPR activation system were electroporated into our clonal GnRH-TdT reporter cell line D7 as previously described (28). The plasmids were a gift from the Timo Otonkoski lab. This line D7 was further single-cell sorted, and the clones were treated with activation medium to measure dCas9 expression with RT-PCR. We chose the clone with the highest dCas9 expression and named this activation line D7C6. Two guide RNAs (gRNAs) targeting the *DLX5* gene (29) were cloned into a plasmid containing blasticidin as a selection marker and transfected into the D7C6 line using Lipofectamine (ThermoFisher Scientific, Cat. STEM00015) along with PiggyBac transposase. DLX5 gRNA 1: AAA​ACT​AGT​TGG​ACG​AGT​TA. DLX5 gRNA 2: GGA​TCT​GGT​TCT​ATT​GGC​CA. This D7C6DLX5 line was treated with 15 μg/mL blasticidin (Thermofisher, Finland Cat. R21001) for ten days and subsequently treated with DOXY and TMP to validate *DLX5* gene activation with RT-PCR.

### Real-time PCR

Total RNA was extracted using NucleoSpin RNA Plus (Machery-Nagel, Germany, Cat. 740984) or RNAqueous™-Micro Total RNA Isolation Kit (ThermoFisher Scientific, Cat. AM1931). 1 μg of mRNA was used to generate cDNA using iScript cDNA Synthesis Kit (Bio-Rad, Germany, Cat. 170-8891). Real-time PCRs were run on Biorad CFX-Opus 96 using HOT FIREPol EvaGreen qPCR Mix Plus (Solis BioDyne, Estonia, Cat. 08-25-00001). Primers used are listed in Supplementary Table 2 (see section on Supplementary materials given at the end of the article).

### Immunocytochemistry

Cells were cultured on Matrigel-coated coverslips (Fisher Scientific, Cat. CB00130RAC20MNZ0) and fixed using 4% paraformaldehyde for 15 min at RT. Coverslips were treated with 0.5% Triton X-100 (Sigma, Cat. T8787) for 10 min followed by blocking with BlockAid™ Blocking Solution (ThermoFisher Scientific, Cat. B10710) for 30 min. Coverslips were incubated overnight with primary antibodies at 4°C. Coverslips were washed with 0.1% PBST three times for 5 min each and then incubated with secondary antibody for 2 h at RT. Following another three washes of 5 min, each coverslip was incubated with DAPI to stain nuclei. After one final wash of 5 min, coverslips were mounted on slides using ProLong™ Diamond Antifade Mountant (ThermoFisher Scientific, Cat. P36961).

### Cell-derived matrix protocol

The protocol was followed as previously described (30) with modifications for GnRH neuron differentiations. 35 mm dishes with glass coverslips (Cellvis, USA, Cat: D35-14-1.5GO) were first coated with Matrigel for a minimum of 30 min at 37°C and then washed twice with PBS. These were crosslinked with 1% glutaraldehyde (Sigma, Cat. G5882) for 30 min at RT. Following crosslinking, dishes were washed twice with PBS. Crosslinker was then quenched with 1 M glycine (Fisher Scientific, Cat. G-0800-60) for 20 min at RT, followed by one wash with 1 M glycine and two washes with PBS. Finally, dishes were recoated with Matrigel overnight at 37°C. Next, day 20 cells were split and plated 1:8 onto the coverslip. Two hours after seeding, medium was added to cover the dish. DAPT medium was refreshed on day 21 and day 23. On day 25, cells were gently denuded using 20 mM NH4OH (Sigma, Cat. 221228), and 0.5% Triton X-100 in PBS for 1 min followed by gently washing cells with PBS. Dishes were immediately fixed in 4% PFA for 20 min and then stored in PBS for staining. Staining protocol was followed as outlined above, omitting the step of permeabilizing with Triton X-100, with Anti-Collagen I + Collagen III antibody (Abcam, Netherlands, ab34710).

### Flow cytometry

Cells were dissociated into single cells with Accutase (NPCs, day 14/15) (Gibco, Finland Cat. A1110501) or papain (neurons, day 25) (Stemcell Technologies, Cat. 07466). Cells were first resuspended in 10% FBS–PBS and pelleted to remove dissociation reagent. Cells were then resuspended in FACS buffer (HBSS, 0.5 mM EDTA, 1 M HEPES, 10% (v/v) FBS, and 10 μM Y-27632), and sorting was performed.

### Wound healing

On day 10 of the differentiation, cells were split 1:2 (refer method 1) and plated on Matrigel-coated Incucyte® ImageLock 96-well plate (Sartorius, Germany, Cat. BA-04855) using three wells for each condition (technical triplicates). Three different doses of FGF8b (100 ng/mL, 25 ng/mL, and no FGF8b) were used. Half of the wells were treated with *DLX5* activation medium and half with normal neuron basal medium. All empty wells were filled with PBS. On day 14, we used Incucyte® 96-Well Woundmaker Tool to generate a horizontal scratch in the wells of the plate. Cells were then washed once with PBS. Fresh medium was added (either activation medium or basal medium), and the plate was scanned every hour in the Incucyte S3 (live-cell imaging and analysis system). We imaged the plate in the Incucyte S3 every hour for 47 h. Analysis was performed using the Scratch Wound Cell Migration & Invasion Assay where relative wound density (RWD) was calculated over 47 h.

### EdU proliferation assay

Proliferation assay for NPCs (day 15) was carried out using Click-iT™ EdU Pacific Blue™ Flow Cytometry Assay Kit (Cat. C10418). Cells were first treated with EdU 4 h prior to collecting the cells. Control cells were left untreated. Cells were dissociated using Accutase for 5 min at 37°C and pelleted, following which the protocol from the kit was performed.

### ELISA

On day 25, we collected medium from *DLX5*-activated and not-activated neurons across all doses of FGF8b to measure secreted GnRH decapeptide. LH-RH/Gn-RH Fluorescent EIA Kit (Phoenix Pharmaceuticals, USA, FEK-040-02) was used following the manufacturer’s instructions. Basal medium was used to normalize the values.

### NeuroTrack analysis

On day 20, NPCs were split 1:8 into 12-well plates (Greiner Bio-One, Austria, Cat no. 665180), three wells for each condition (technical triplicates). Four different conditions were measured for TdT+ neurons: i) no FGF8b, no activation; ii) no FGF8b, *DLX5* activation (days 10–20); iii) 100 ng FGF8b, no activation; and iv) 100 ng FGF8b, *DLX5* activation (days 10–20). On day 23, after refreshing the cells with fresh DAPT medium, the 12-well plate was placed in Incucyte® and the neurons were imaged for 48 h. Data were analyzed using the Incucyte® NeuroTrack software module, which allows users to study neurite and nuclei parameters. The nuclear TdT reporter in the GnRH neurons was used to calculate the number of red cells within the well. This was calculated by the metric ‘average area red’, which measures the number of objects identified with red fluorescence channel in a given area (μm^2^).

### RNA-sequencing and bioinformatics pipeline

Total RNA was extracted using NucleoSpin RNA (Machery-Nagel, Germany, Cat. 740955.250). Samples were sent to Novogene GmbH, Munich, Germany, for sequencing and bioinformatics. Poly-T-oligo attached beads were used to isolate messenger RNA (mRNA) from total RNA. Following purification, the mRNA was subjected to fragmentation for subsequent cDNA synthesis. The synthesis of the first strand of complementary DNA (cDNA) was conducted using random hexamer primers. For directional libraries, dUTP was incorporated, allowing for strand-specific sequencing, while non-directional libraries utilized dTTP. The initial stage of the bioinformatics analysis involved quality control of the raw sequencing data. The raw reads, in fastq format, were processed using the fastp software. The quality of the clean data was further assessed by calculating Q20, Q30, and GC content metrics. Mapping of the clean reads to a reference genome was performed using Hisat2 v2.0.5. The quantification of gene expression levels was conducted using featureCounts v1.5.0-p3. This tool facilitated the counting of reads mapped to each gene. For datasets with biological replicates, DESeq2 (version 1.20.0) was employed. DESeq2 uses a model based on the negative binomial distribution to determine differential expression in digital gene expression data. *P*-values were adjusted using the Benjamini and Hochberg method to control the false discovery rate. Genes with an adjusted *P*-value of <0.05 were classified as differentially expressed. Enrichment analysis of differentially expressed genes was carried out using the clusterProfiler R package. Gene ontology (GO) enrichment analysis was implemented with corrections for gene length bias. GO terms with a corrected *P*-value of less than 0.05 were considered significantly enriched.

### Artificial intelligence (AI)

We acknowledge Claude Opus 4.7 (Anthropic) for assistance in language editing and text improvement.

## Results

### Generation of *DLX5* CRISPR activation line

**Figure 1:**
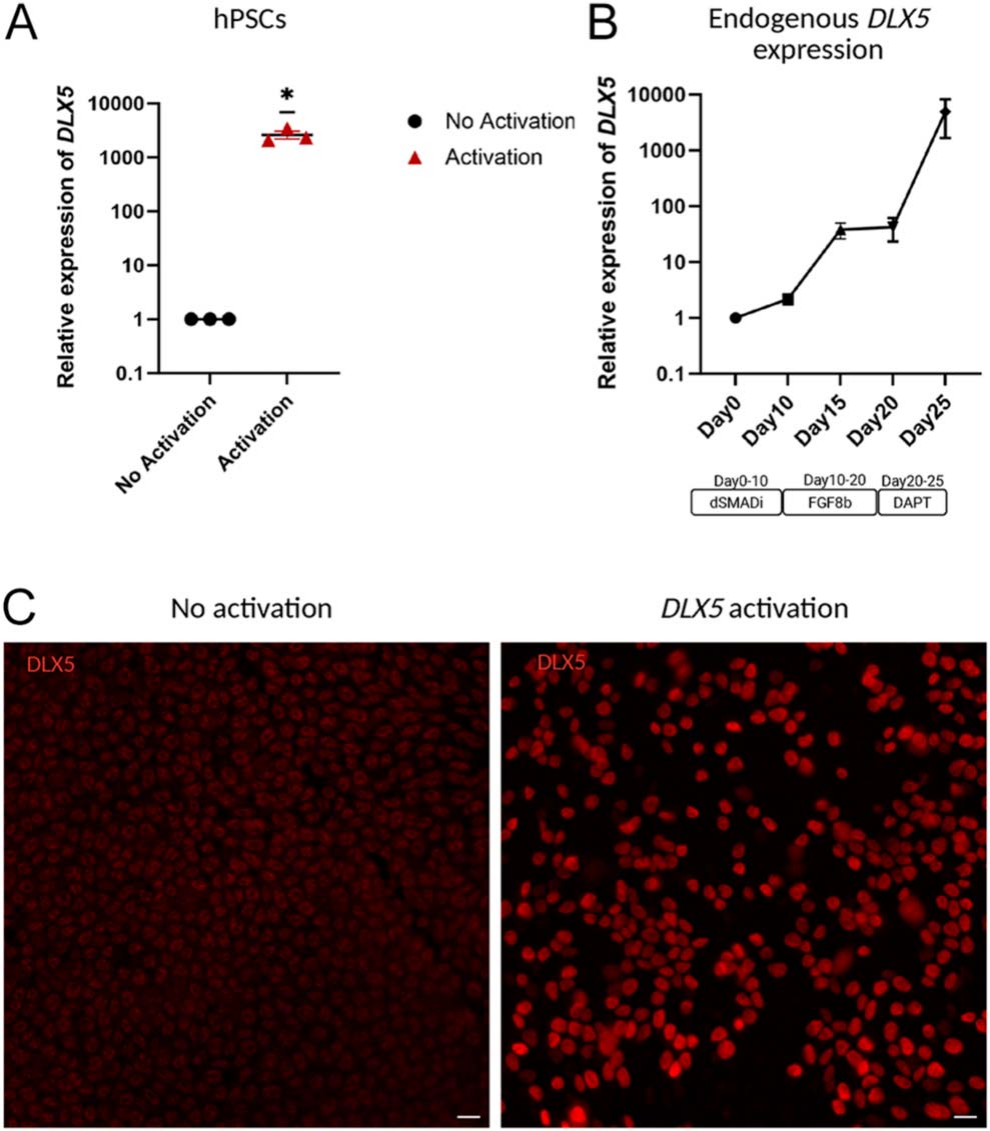
(A) RT-PCR shows increased *DLX5* mRNA expression in hPSCs after 48 h of activation. *n* = 3, *P < 0.05 with a one-sample *t*-test. The horizontal lines represent arithmetic mean and ± SEM. (B) Expression of endogenous *DLX5* mRNA across the hPSC-derived GnRH neuron differentiation protocol. (C) A representative immunofluorescence image showing increased DLX5 protein expression in hPSCs after 48 h of activation. Scale bar, 1 µm. A full color version of this figure is available at https://doi.org/10.1530/JME-26-0040.

We generated a clonal *DLX5* CRISPR activation line by electroporating the DDdCas9VP192 system (28) and two gRNAs (29) targeting the first exon of the *DLX5* gene. Both *DLX5* mRNA and protein levels were significantly upregulated compared to not-activated control hPSCs when treated with activation medium (Fig. 1A and C). In addition, we checked the endogenous expression of *DLX5* mRNA throughout the hPSC-derived GnRH neuron differentiation protocol (Fig. 1B), which showed a trend of increasing expression toward the later neural progenitor and neuron stages. Therefore, the hPSC *DLX5* activation line provides a robust model to study the effect of *DLX5* overexpression.

### *DLX5* activation promotes motility of GnRH neural progenitor cells independently of FGF8b dose

**Figure 2:**
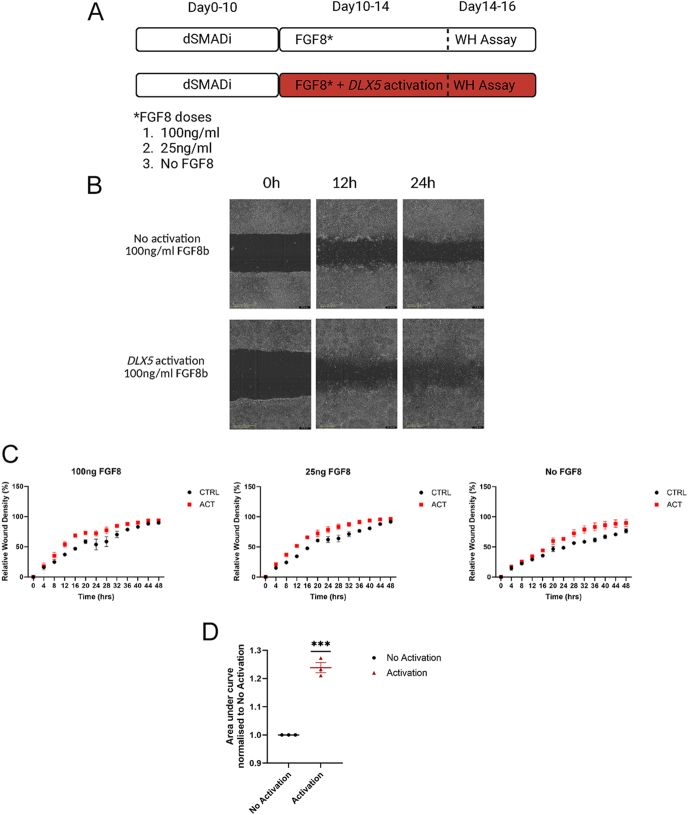
(A) Schematic of our hPSC-derived GnRH neuron differentiation protocol illustrating the phase of *DLX5* activation (days 10–16) and FGF8b doses. (B) Bright-field images showing the relative wound density over time at 0 h, 12, and 24 h (cells treated with 100 ng/mL FGF8b from days 10 to 16). The bottom panel shows cells that were activated. (C) Graphs showing an increase in relative wound density over 47 h for each of the three FGF8b doses (*n* = 3). (D) Area under curve was calculated comparing all the not-activated samples and the activated samples, across all FGF8b doses (*n* = 3), one-sample *t*-test ***P < 0.001. The horizontal lines represent arithmetic mean and ± SEM. A full color version of this figure is available at https://doi.org/10.1530/JME-26-0040.

Previously, we have shown that 25 ng/mL of FGF8b is adequate to generate GnRH neurons from hPSCs (31). To investigate the crosstalk of *DLX5* activation and *FGF8* signaling, we used 100 ng/mL, 25 ng/mL, and no FGF8b conditions (Fig. 2A). We hypothesized that activation of *DLX5* may promote motility of NPCs (21) and tested this with a wound-healing assay from days 14 to 16 (Fig. 2B), where we activated *DLX5* from days 10 to 16 (Fig. 2A). We observed across the three FGF8b doses that the rate at which the cells closed the wound (RWD – relative wound density, over 47 h) was higher in the activated conditions (Fig. 2C). The area under curve analysis from RWD (Fig. 2C) showed that *DLX5*-activated cells closed the gap significantly faster compared to cells that were not activated (Fig. 2D). We conducted an EdU proliferation assay to assess whether the observed increase in wound closure was due to proliferation rather than increased motility. We show that the proliferation rates were not significantly different between *DLX5*-activated and not-activated conditions (Supplementary Fig. S1A). This suggests that the enhanced wound healing observed in *DLX5*-activated cells is primarily due to increased motility.

### *DLX5* activation augments the GnRH neuron population and can rescue *GNRH1* expression even in the absence of FGF8b

**Figure 3:**
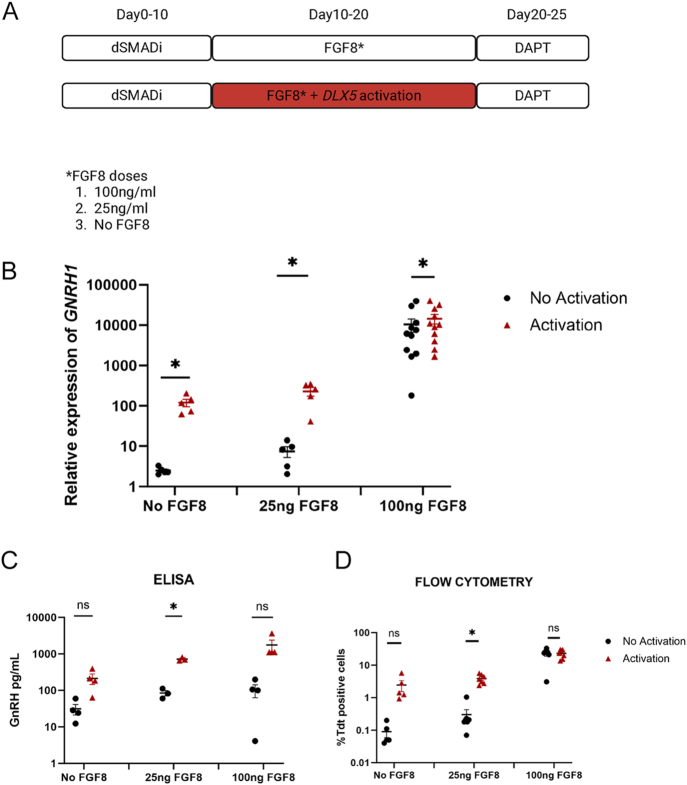
(A) Schematic of hPSC-derived GnRH neuron differentiation protocol illustrating the phase of *DLX5* activation (days 10–20) and FGF8b doses. (B) RT-PCR of *GNRH1* comparing day 25 *DLX5*-activated (days 10–20) neurons to that of not-activated controls across different doses of FGF8b. No FGF8b (*n* = 5), 25 ng/mL FGF8b (*n* = 5), and 100 ng/mL FGF8b (*n* = 11). Paired *t*-tests show significance in all conditions. *P < 0.05. (C) ELISA showing GNRH decapeptide secretion from day 25 neurons across three FGF8b doses comparing not-activated controls to *DLX5*-activated (days 10–20) neurons. No FGF8b (*n* = 4), 25 ng/mL FGF8b (*n* = 3), and 100 ng/mL FGF8b (*n* = 4). A paired *t*-test shows significance only in the 25 ng/mL condition. *P < 0.05. (D) Flow cytometry results showing percentage of day 25 TdT+ neurons comparing not-activated controls and *DLX5*-activated (days 10–20) neurons. No FGF8b (*n* = 5), 25 ng/mL FGF8b (*n* = 7), and 100 ng/mL FGF8b (*n* = 7). A paired *t*-test shows significance only in the 25 ng/mL condition. *P < 0.05. In no FGF8b conditions in (B and C), *P* = 0.05. The horizontal lines represent arithmetic mean and ± SEM. A full color version of this figure is available at https://doi.org/10.1530/JME-26-0040.

Our previously published single-cell transcriptomics data demonstrate that *DLX5* expression precedes that of *GNRH1* (27). Therefore, we assessed *GNRH1* expression on day 25 after activation of *DLX5* during the FGF8b stage of our protocol (days 10–20) across three doses of FGF8b (Fig. 3A). Interestingly, we found that activation of *DLX5* with 100 ng/mL of FGF8b significantly increased *GNRH1* expression on day 25 (Fig. 3B). Activation of *DLX5* was able to rescue *GNRH1* expression in the 25 ng/mL FGF8b and no FGF8b conditions unlike the not-activated controls (Fig. 3B). Incucyte NeuroTrack live imaging analysis showed more GnRH-TdT neurons, measured by Average Area Red (μm^2^), in the *DLX5*-activated cells on day 25 in both no FGF8b and 100 ng/mL FGF8b (Supplementary Fig. S1B) conditions. ELISA also showed significantly increased secretion of GnRH decapeptide in *DLX5-*activated GnRH neurons for 25 ng/mL dose (Fig. 3C). Flow cytometry analysis of TdT neurons showed a significant increase in the *DLX5*-activated cells with 25 ng/mL FGF8b and borderline statistical significance in the no FGF8b condition (*P* = 0.05) (Fig. 3D). We measured the expression of endogenous *FGF8* mRNA on day 20 cells across two FGF8b doses (no FGF8b and 25 ng/mL) and observed a significant increase in *FGF8* in both the *DLX5*-activated conditions (Supplementary Fig. S1C).

### *DLX5-*activated cells exhibit increased expression of genes associated with neural migration and extracellular matrix formation

We conducted bulk RNA-sequencing on day 20 NPCs and day 25 neurons treated with 100 ng/mL FGF8b to assess the transcriptional changes associated with *DLX5* activation (Supplementary Fig. S1D). In all conditions, we compared the transcriptomes of *DLX5*-activated cells to corresponding not-activated controls. Genes that had padjust < 0.05, log2fold changes ≥1, and read counts ≥95 were considered differentially expressed genes (DEGs). Assessment of the associated GO terms revealed that the most significant biological process in day 20 and day 25 total cell populations was promoting formation of the extracellular matrix (ECM) (Table 1, Supplementary Fig. S2). In the *DLX5*-activated conditions, expression of many genes from the collagen family (*COL1A1, COL1A2, COL6A2, COL5A1*) along with genes (*MMP1, MMP2, ADAMTs*) encoding matrix metalloproteinases that interact with basic ECM components (32) was upregulated. Another GO term of interest across all the datasets was axon guidance (Supplementary Fig. S2), with several associated genes important for GnRH neuron migration, including *SEMA3E* (33), *DCC* (34), and *RELN* (35) (Table 1). Unexpectedly, we also observed other hypothalamic neuron markers, such as *KISS1R* and *POMC*, upregulated by *DLX5* activation on day 20 and *TAC3* in the day 25 neurons, suggesting accelerated maturation of neurons.

**Table 1:** Bulk RNA-sequencing data from three lists of differentially expressed upregulated genes. All data sets compare *DLX5*-activated cells (days 10–20) versus not-activated cells treated with 100 ng/mL FGF8b; therefore, genes are upregulated when *DLX5* is activated. Column 1 indicates important biological processes chosen from the top 20 GO terms unless otherwise indicated. Column 2 shows genes from day 20 NPCs. Column 3 indicates genes from day 25 neurons (all GnRH-TdT+ neurons and TdT-negative neurons). Column 4 indicates genes from day 25 TdT+ cells only after FACs. Genes of interest in this study are indicated in bold.

Biological process	Day 20 NPCs	Day 25 pooled neurons	Day 25 TdT + neurons
Axon guidance	PRKCQ, SPTBN4, ALCAM, NRP1, LAMA2, NRXN3, SH3KBP1, SPTB, **WNT5A**, **SEMA3C**, GFRA1, **RELN**, SLIT3, NGFR, NRTN, GFRA2, EFNA1, PTPRO, RAP1GAP, CNTN4, **UNC5C, DCC, PLXNA4**, CXCL12, **DSCAM**, DLX5, **SEMA3E**	**WNT5A**, MATN2, LAMA2, LAMB2, NFIB, CNTN4, VAX1, ETV1, CXCR4, EFNA4, GLI2, SEMA5A, UNC5C, UNC5B, GLI3, BMP7, FLRT2, ALCAM, BOC *	GFRA2, **SEMA5A**, **UNC5C**, BCL11B, **RELN**, PAX6, VEGFA, PLXNA2, **DSCAM**, PTPRM, TNR, **PLXNA4**, ISL1
ECM related	SERPINF1, EGFL7, BCAM, TGFB1, EFEMP1, CDON, TGFB3, LRRN2, FLRT1, COL9A3, ALPL, FBLN1, PCOLCE, EMILIN1, ADAMTS10, SSC5D, NID2, FBLN5, LINGO3, ANGPTL4, LAMA2, SOD3, CILP2, ADAMTS2, WNT5A, COL11A2, TGFBI, **LAMB1**, RELN, SLIT3, COL14A1, CD248, COL6A2, ECM1, CSPG4, FREM2, SMOC1, RARRES2, FBLN2, WNT11, ADAMTSL4, ADAMTSL2, BGN, ICAM1, HMCN2, SLITRK6, CTHRC1, CRISPLD2, MFAP4, TGM2, COL1A2, SPON1, LRFN2, ACAN, COL8A2, GDF10, MMP1	**SPARC**, BGN, POSTN, WNT5A, COL1A2, COL1A1, DCN, NID1, CD248, BMP4, MMP14, COL5A1, TGFBI, SPON1, TIMP3, NDNF, HMCN1, COL8A1, **LAMB1**, FBN2, COL6A2, MATN2, PCOLCE, COL3A1, EMILIN3, COL2A1, FBLN2, FREM2, LTBP1, TNC, WNT9B, WNT8B, MFGE8, LAMA2, COL22A1, COL5A2, COL18A1, LAMB2, COL27A1, LOX, MFAP4, COL9A3, COL9A1, FBN1, EMILIN1, HSPG2, FGFR2, FN1, LTBP3, P3H2, AMBN, CYR61, CRTAP, THBS1, SMOC1, AEBP1, ALPL, COL4A1, HTRA1, MFAP2, BMP7, TGFB1, FLRT2, TGFB2, COL11A1, COL4A5, ELN, BCAM, LRFN5, APOE, MMP2, LGALS3BP, LTBP4, FRAS1, EFEMP2, LOXL2	COL1A2, LRFN5, GPC3, MFGE8, SFRP2, RELN, VEGFA, PCSK6, TNR, SPOCK2, **LAMB1**, **LINGO2**, GPC6 **
GnRH neuron related***	**GAD2**, POMC, FGFR4, KISS1R, **GAD1**, RBFOX1	TAC3, NDNF, ISLR	SST, SSTR1
DLX related***	DLX5, DLX4, DLX3, DLX6, DLX2, MSX1, DLX1	MSX2, MSX1	MSX1

* Axon guidance GO term was ranked 717 in the list;

** ECM GO term was ranked 62 in the list;

*** Categories chosen to focus on genes of interest to this paper.

### Expression of genes associated with axon guidance is altered in GnRH neurons generated by *DLX5* activation in the absence of FGF8b

**Figure 4:**
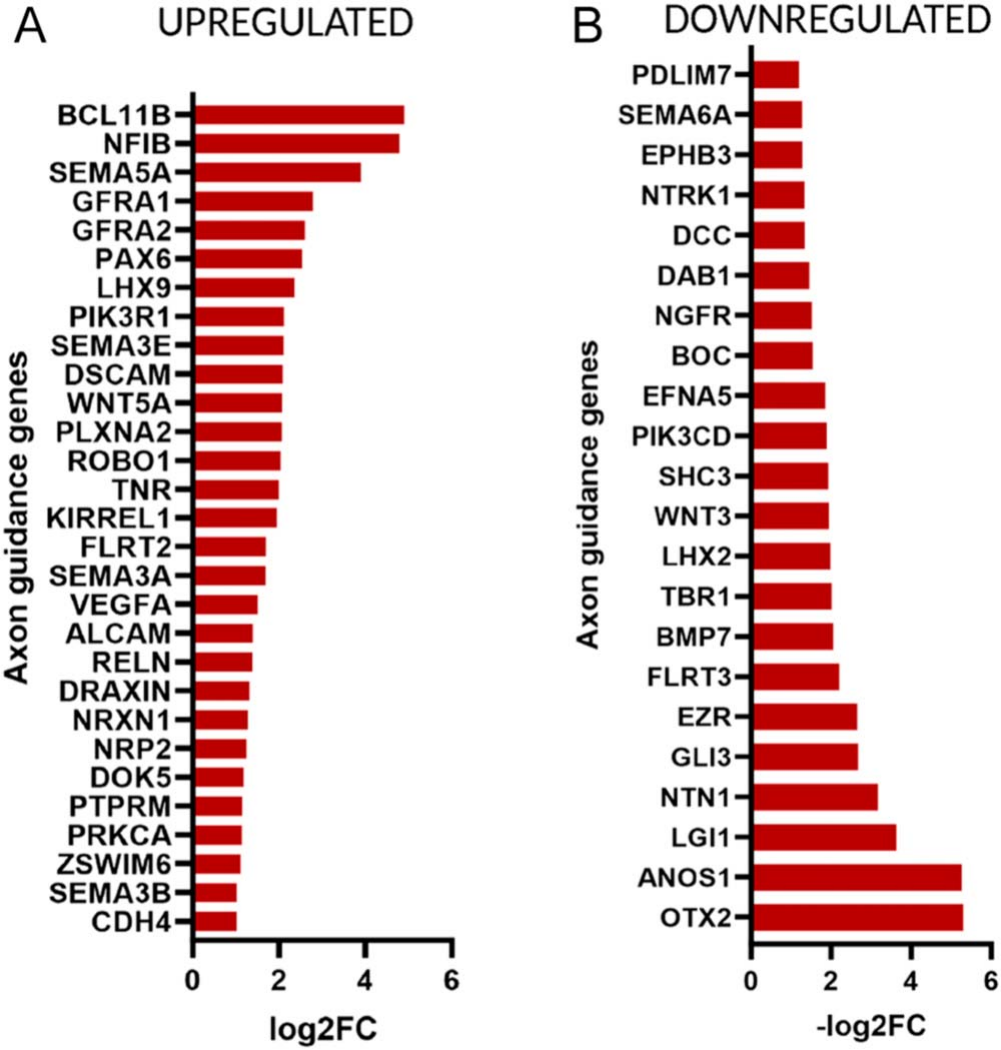
Axon guidance genes ordered by log2fold changes from top 20 GO terms comparing day 25 no FGF8b *DLX5*-activated GNRH-TdT neurons to 100 ng/mL FGF8b not-activated GNRH-TdT neurons. (A) Upregulated genes. (B) Downregulated genes. A full color version of this figure is available at https://doi.org/10.1530/JME-26-0040.

*DLX5* activation was able to direct a population of NPCs to GnRH neurons in the absence of exogenous FGF8b (Fig. 3B). We FACs-sorted and sequenced day 25 *DLX5-*activated GnRH-TdT neurons and compared the transcriptomic profiles of the cells generated by *DLX5* activation alone (without FGF8b) to those generated by treatment with 100 ng/mL exogenous FGF8b (Supplementary Fig. S1E). From the GO term analysis, axon guidance was in the top 20 in both the upregulated and downregulated lists (Supplementary Fig. S3). The upregulated gene list (Fig. 4A) included genes such as *SEMA5A, SEMA3E, SEMA3A, SEMA3B, DSCAM*, *PLXNA2,* and *RELN,* all of which contribute toward axon guidance. Conversely, in the downregulated list, known markers that aid GnRH neuron migration, such as *DCC* (36, 37) and *ANOS1* (38) (Fig. 4B), were differentially expressed. Interestingly, *SEMA6A,* which regulates innervation of GnRH neurons into the median eminence (39), was also downregulated.

### *DLX5* activation leads to a distinct spatial patterning effect and increases expression of extracellular matrix markers such as *SPARC* and collagen

**Figure 5:**
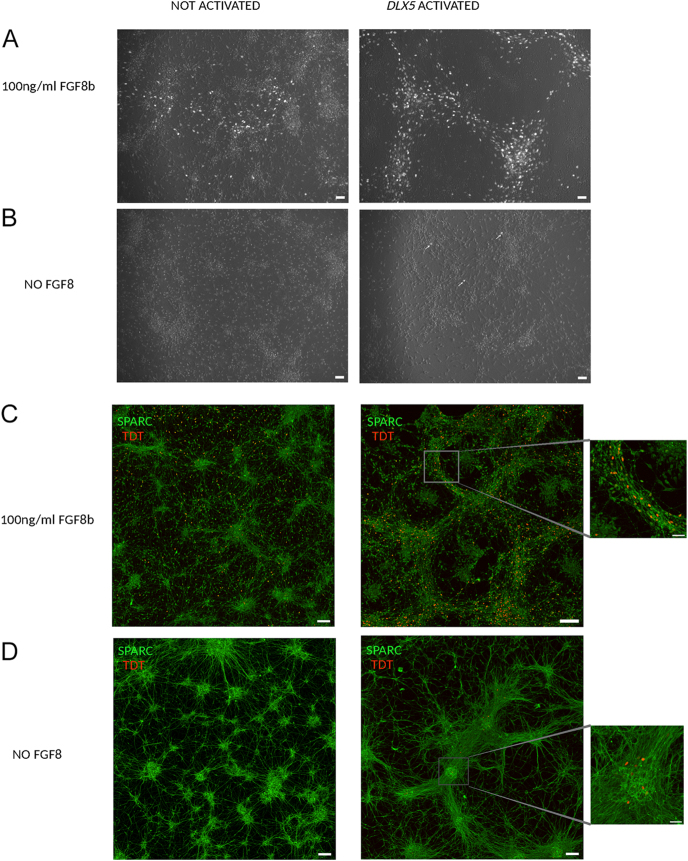
All images on the left panels represent not-activated conditions. All images on the right panels represent *DLX5-*activated conditions. (A) Day 25 TdT+ neurons that were treated with 100 ng/mL FGF8b (days 10–20). (B) Day 25 TdT+ neurons (marked with white arrows) not treated with FGF8b (days 10–20). (C and D) Representative immunofluorescence images with an antibody specific to SPARC in day 25 GNRH-TdT+ neurons. (C) Neurons that were treated with 100 ng/mL exogenous FGF8b between days 10 and 20. (D) Neurons without FGF8b. Scale bars: 100 µm. A full color version of this figure is available at https://doi.org/10.1530/JME-26-0040.

**Figure 6:**
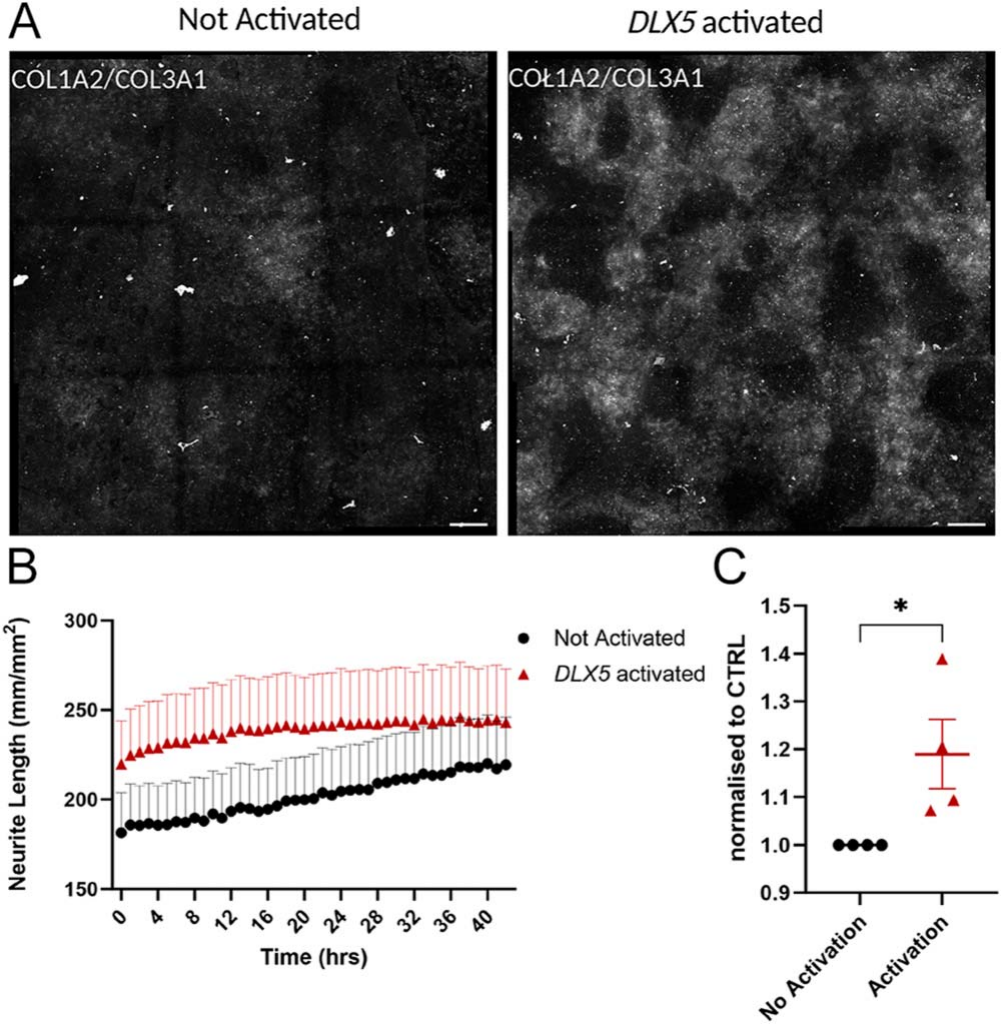
(A) Representative immunofluorescence images of ECM collagen from cell-derived matrices from denuded day 25 GNRH-TdT+ neurons treated with 100 ng/mL FGF8b (days 10–20). The left panel shows not-activated conditions, and the right panels show *DLX5*-activated condition. Scale bar: 100 μm. (B) Neurite lengths from days 23 to 25 neurons, *n* = 4. (C) Area under curve for the data shown in (B), data analyzed with unpaired *t*-test, *P < 0.05. The horizontal lines represent arithmetic mean and ± SEM. A full color version of this figure is available at https://doi.org/10.1530/JME-26-0040.

Interestingly, simultaneous *DLX5* activation and 100 ng/mL FGF8b treatment resulted in a distinct spatial organization/patterning of the day 25 neurons (Fig. 5A, Supplementary Fig. S4). GnRH neurons started to aggregate and form distinct tracts in a honeycomb-like structure. We did not observe this phenomenon in the absence of FGF8b (Fig. 5B). Looking into the single-cell RNA-sequencing data previously published (27), we hypothesized that perhaps *DLX5* is promoting the non-proliferating progenitor pool (NNP), and these are the cells that facilitate the honeycomb structure. The NNP cluster comprises cells expressing both progenitor and neural markers. Comparison of the pooled day 25 bulk RNA-seq data and the single-cell NNP cluster data revealed a modest overlap of genes. Indeed, among the overlapping genes, we identified *SPARC*, a gene encoding matrix-associated protein (40, 41). Immunofluorescence of day 25 cells showed the ECM marker SPARC inside and around the honeycomb structure (Fig. 5C and D). In addition, from bulk RNA-sequencing data across day 20 and day 25, several collagen markers were upregulated in the *DLX5*-activated cells (Table 1). On day 25, under the *DLX5*-activated cells, collagen was aggregated in a similar honeycomb pattern as TdT-expressing neurons (Fig. 6A). Finally, neurites of the *DLX5*-activated neurons (treated with 100 ng/mL FGF8b) were significantly longer than those of their not-activated counterparts (Fig. 6B and C).

## Discussion

The role of *DLX5* in regulating axon guidance cues, ECM organization, and the spatial patterning of GnRH neurons in humans remains largely unexplored. In mice, *Dlx5* is expressed from the earliest stages of olfactory development, where it has been detected across multiple compartments of the peripheral and central olfactory system, including the OPs, neuroepithelium, VNO, and OBs (9, 21, 22, 42). In addition, *Dlx5* and *Gnrh1* are co-expressed in mouse migratory cells exiting the OPs (21), whereas the role of *DLX5* in human GnRH neuron ontogeny is unclear. Migrating human GnRH neurons co-express *DLX5,* and in hPSC-derived GnRH neurons, *DLX5* expression precedes that of *GNRH1* (27). In the current work, we examined whether *DLX5* modulates the differentiation of GnRH neurons from hPSCs.

We first probed the question of whether activation of *DLX5* affects NPC motility. To achieve this, we activated *DLX5* expression during the NPC (FGF8b) phase with CRISPR activation and measured the rate of wound healing (relative wound density). Interestingly, *DLX5* activation resulted in faster closure of the wound in NPCs, and this was not due to increased proliferation. Similarly, in mice, *Dlx5* appears not to affect neural stem cell proliferation (43). We next considered possible mechanisms that could lead to increased motility of the NPCs. Indeed, we observed that on day 20, representing the end of NPC phase, *DLX5* activation induced several genes required for cell migration, adhesion, axon guidance, survival, and synapse formation, such as *SEMA3C, SEMA3E, PLXNA4, UNC5C, DCC,* and *DSCAM* (44, 45). Notably, *SEMA3C* is an axon attractant cue (46) and variants in *SEMA3E* cause normosmic hypogonadotropic hypogonadism/Kallmann syndrome (47, 48), which underscores their importance in GnRH neuron biology. Cariboni and colleagues also show that *SEMA3E* is important for the survival of GnRH neurons (33). *SEMA6D–PLXNA4* signaling in the amygdala regulates synapse maturation and GABAergic signaling (49). Notably, several semaphorins (*SEMA5A, SEMA3A, SEMA3B, SEMA3E)* were upregulated in *DLX5*-activated hPSC-derived GnRH-TdT+ neurons, an observation consistent with previous reports in murine models (50, 51, 52) warranting further investigation. Moreover, the absence of exogenous FGF8b did not affect the expression of semaphorins, suggesting that *DLX5* activation can promote their expression with or without exogenous FGF8b. We also identified the upregulation of netrin-1 receptors *UNC5C* (53), *DCC* (34, 36), and *DSCAM* (54) that are required for axonal growth cone, axon turning, and axon branching. *RELN*, upregulated by *DLX5* activation on day 20, is important for GnRH neuron migration (35) and for the development of ECM structure in human neocortex (55). These observations suggest that *DLX5* may orchestrate the expression of guidance cues necessary for neuronal pathfinding and connectivity.

*DLX5* overexpression augmented the GnRH neuron pool, and therefore, we hypothesized that *DLX5* may alter cell fate decisions. Transcriptomic analysis revealed that several key GABAergic markers were upregulated following *DLX5* activation, such as *GAD1, GAD2,* and *WNT5A*. In addition, DLX1/2/5 factors have been shown to bind directly to the *Gnrh1* promoter region (7), suggesting that the increased *GNRH1* expression may be due to *DLX5* activation. These findings are consistent with the known roles of Dlx factors in GABAergic forebrain neurogenesis (8, 24), where they co-localize with GAD1 and GAD2, and ectopic expression of *Dlx2* and *Dlx5* induces *Gad2* expression and a GABAergic phenotype in cortical cells (56). *Dlx5*^−/−^ mice show reduced numbers of GABAergic neurons (42), and downregulation of miRNA-9 and miRNA-200 in these mice increases *Foxg1* expression and delays differentiation (57). The mouse ortholog of *Wnt5a* promotes formation of GABAergic olfactory interneurons and is a direct target of DLX5 (58). Collectively, these results suggest that *DLX5* activation in NPCs promotes a GABAergic identity in differentiating NPCs, enhancing GnRH neurogenesis.

*FGF8* is required for GnRH neuron specification (31, 59, 60), but whether *DLX5* can drive *GNRH1* expression independently of exogenous FGF8b has not been established. We show that when *DLX5* is activated in NPCs, there is an increase in both *GNRH1* mRNA expression and secretion of GnRH decapeptide from day 25 neurons even without FGF8b treatment. Several mechanisms may underlie this FGF8b-independent effect. First, *DLX5* activation may generate a larger pool of ‘*DLX5*-committed progenitors’, which could drive *GNRH1* expression in the cells treated with low doses or in the absence of FGF8b. In the mouse olfactory system, *Dlx5* is not expressed in uncommitted mitotic NPCs but in a subset of committed NPCs (43). Consistent with this, upregulation of *Dlx5* and *Dlx6* prevents cells from entering S-phase and promotes neural differentiation (8, 61). Second, *DLX5* is an upstream regulator of *FGF8* (62, 63)*,* and we show that *DLX5* overexpression induces endogenous *FGF8* expression*.* These data therefore demonstrate that *DLX5* can promote GnRH neuron identity through multiple convergent pathways, partially bypassing the requirement for exogenous FGF8b.

Beyond transcriptomic changes, we observed that the combined action of *DLX5* and FGF8b also had notable structural consequences on neuronal cultures, resulting in a distinct honeycomb-like spatial patterning of the day 25 TdT+ neurons and an increase in neurite length. *DLX5* activation upregulated the expression of *SPARC, MMP1, MMP2,* and *LINGO2* in day 25 neurons. *SPARC,* secreted by olfactory ensheathing cells, functions in the promotion of cell survival, modifies cell adhesion and migration, regulates laminin-1 and TGF-beta signaling to stimulate Schwann cells to promote neurite outgrowth, and interestingly may also regulate the production of collagen (64, 65). Matrix metalloproteinases MMP1 and MMP2 function by regulating the amount of ECM and preventing it from overgrowing (32). Interestingly, *lingo2* is a putative target of *Dlx5* and depleting *lingo2* in zebrafish led to minor defects in the organization and shape of the OPs (66). *Dlx5^−/−^* mice exhibit disorganized cellular layers in OBs, indicating that *DLX5* may indeed orchestrate cellular arrangement (42). These findings suggest that *DLX5* coordinates the expression of ECM components that together shape the migratory landscape and spatial organization of developing GnRH neurons.

Pathogenic variants in *DLX5* and/or *DLX5/DLX6* lead to split-hand/foot malformation (SHFM) (63, 67), characterized by limb malformations and missing/claw-like digits. However, there is currently no documented evidence of GnRH deficiency in patients carrying pathogenic *DLX5* variants. In addition, there is functional redundancy and regulatory compensation among the DLX family genes (23, 68, 69), and therefore, we focused on *DLX5* activation. In conclusion, the present study investigates the role of *DLX5* during the differentiation of hPSC-derived GnRH neurons. Activating *DLX5* expression in NPCs increased their motility, augmented subsequent emergence of GnRH neurons, and upregulated expression of genes associated with axon guidance, GABAergic neurons, and ECM components.

## Supplementary materials











## Declaration of interest

The authors declare that there is no conflict of interest that could be perceived as prejudicing the impartiality of the work reported.

## Funding

This study was financially supported by Research Council of Finland Academy Research Fellowship (315,616 to TR; 1,360,775 to JRWC), Sigrid Juselius Foundation (TR), Foundation for Pediatric Research (TR), The Hospital District of Helsinki and Uusimaa/Children and Adolescents (TR), European Union’s Horizon 2020 research and innovation program under the Horizon 2020 Framework Programme Marie Sklodowska-Curie Actions grant agreement 813707 (SM), The Vilho, Yrjö and Kalle Väisälä Foundation (SM and NE), and Instrumentarium Science Foundation (SM and CGS).

## Author contribution statement

SM conceived the study, performed the experiments, analyzed the data, acquired funding, and wrote, reviewed, and edited the manuscript. NE and CGS performed the experiments and reviewed and edited the manuscript. KP wrote, reviewed, and edited the manuscript. KV performed the experiments, managed the project, acquired funding, and reviewed and edited the manuscript. VY performed the experiments (CRISPR work). JRWC performed the experiments (CDM) and edited the manuscript. YW conceived the study. TR conceived the study, administered the project, supervised the study, and acquired funding, and wrote, reviewed, and edited the manuscript. All authors read and approved of the final manuscript.

## Data availability

All bulk RNA-sequencing data related to the manuscript are available on Gene Expression Omnibus with accession ID: GSE337839. All other data are available on request from the corresponding author.
