# Supplementary figures and images for: CRISPR activation of DLX5 drives neural progenitors to the GnRH cell fate
Source: J Mol Endocrinol. 2026 Jul 23;77(1):e260040. doi: 10.1530/JME-26-0040 (PMC13400967; doi:10.1530/JME-26-0040)

1A)

## EdU Proliferation Assay

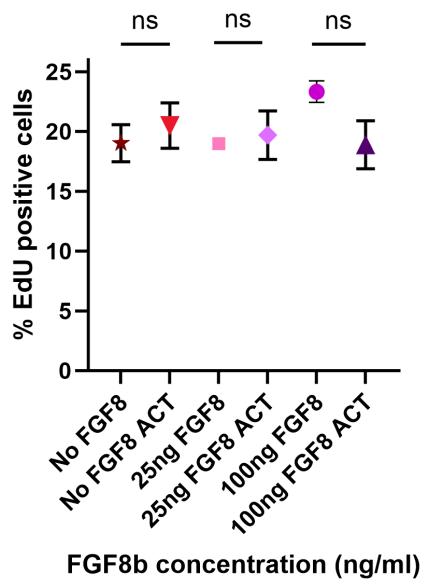

1B)

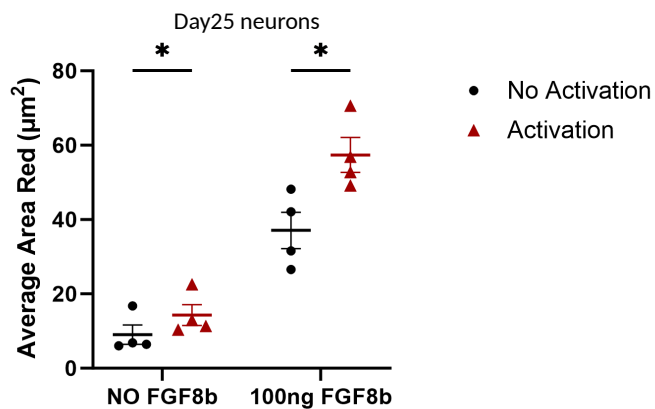

1C)

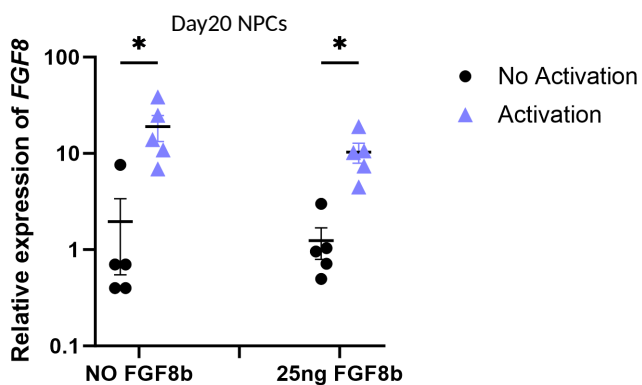

1D)

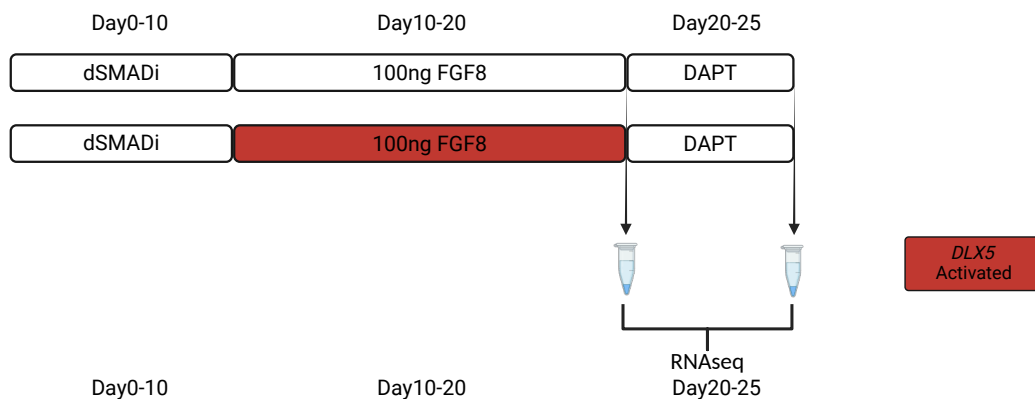

1E)

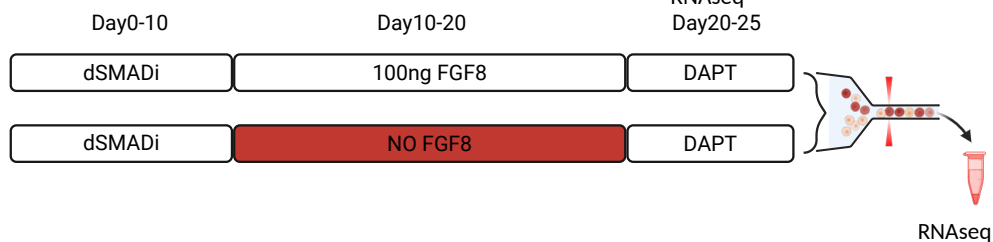

Supplement: Supplementary file 1 [file JME-26-0040_supplementary_figure_1.pdf]

## 2A) Day20 NPCs

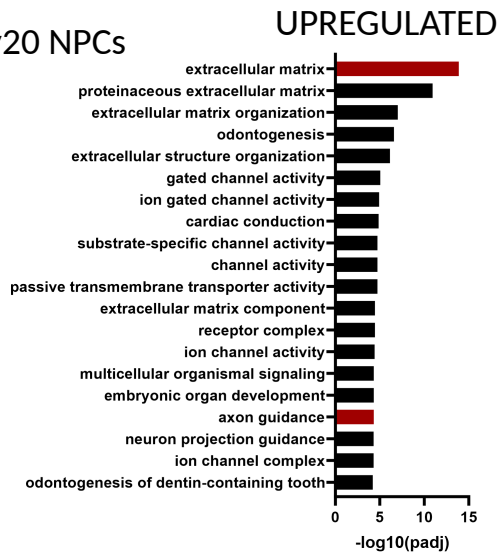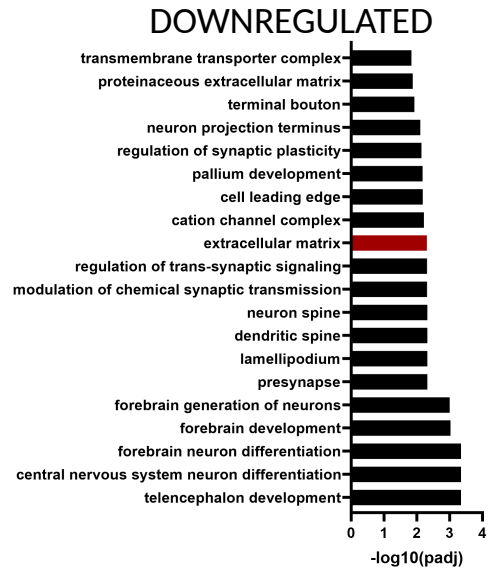

## 2B) Day25 Pooled neurons

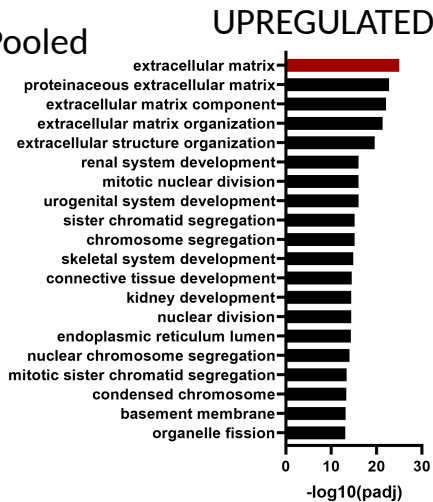

## 2C) Day25 TdT+ neurons

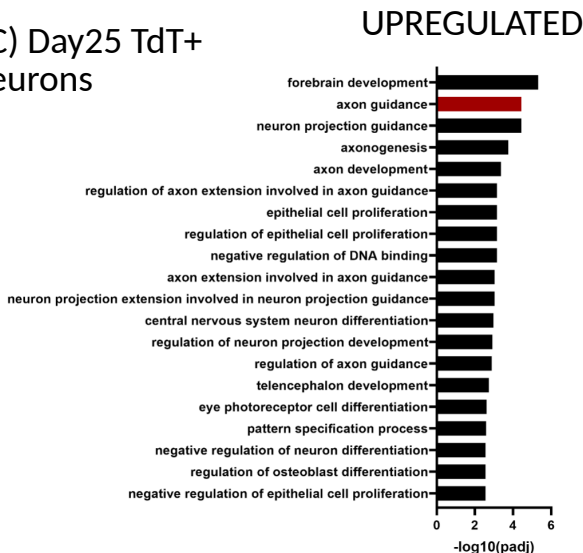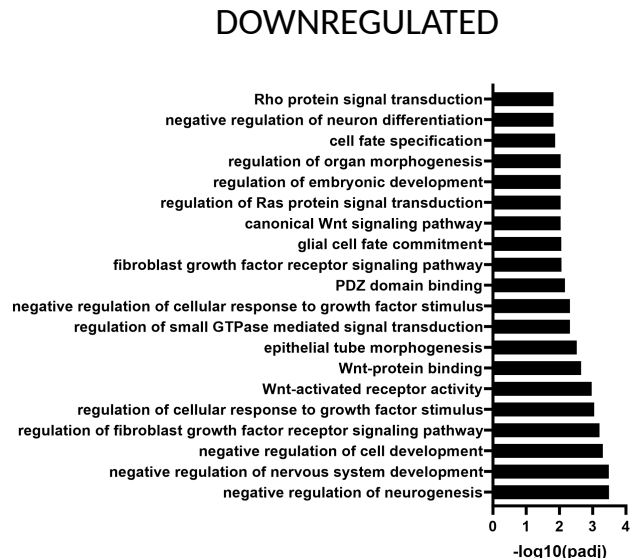

Supplement: Supplementary file 2 [file JME-26-0040_supplementary_figure_2.pdf]

Supplementary Figure S3

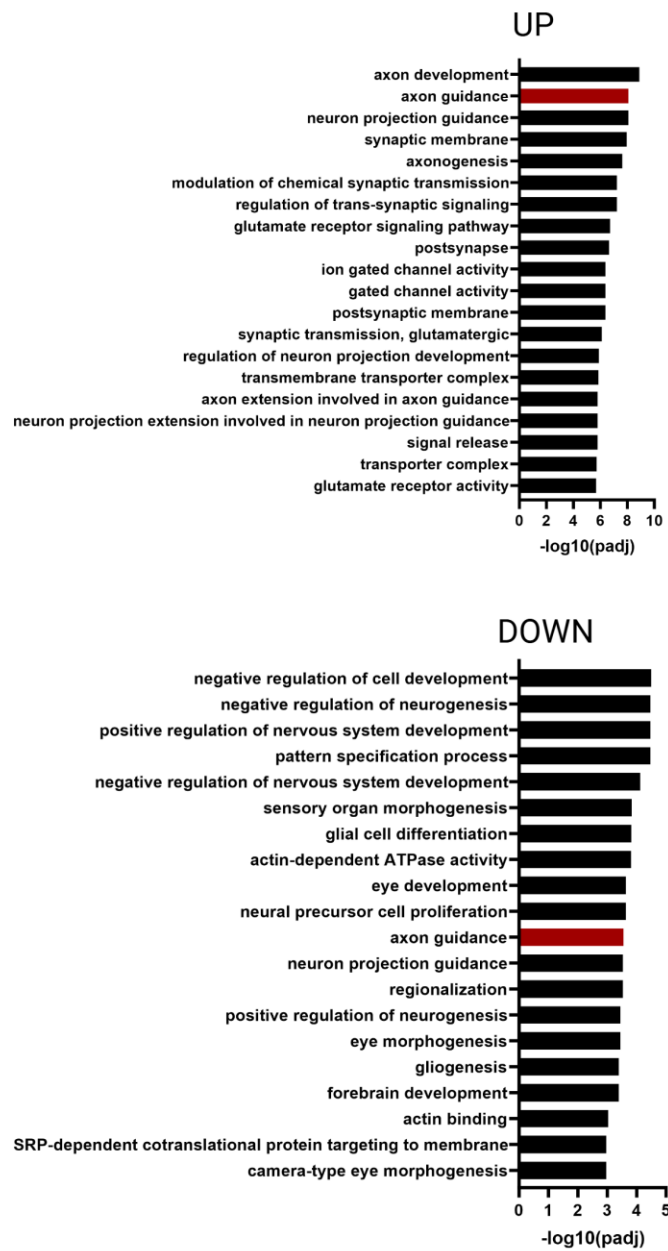

Supplement: Supplementary file 3 [file JME-26-0040_supplementary_figure_3.pdf]

Supplementary Figure S4

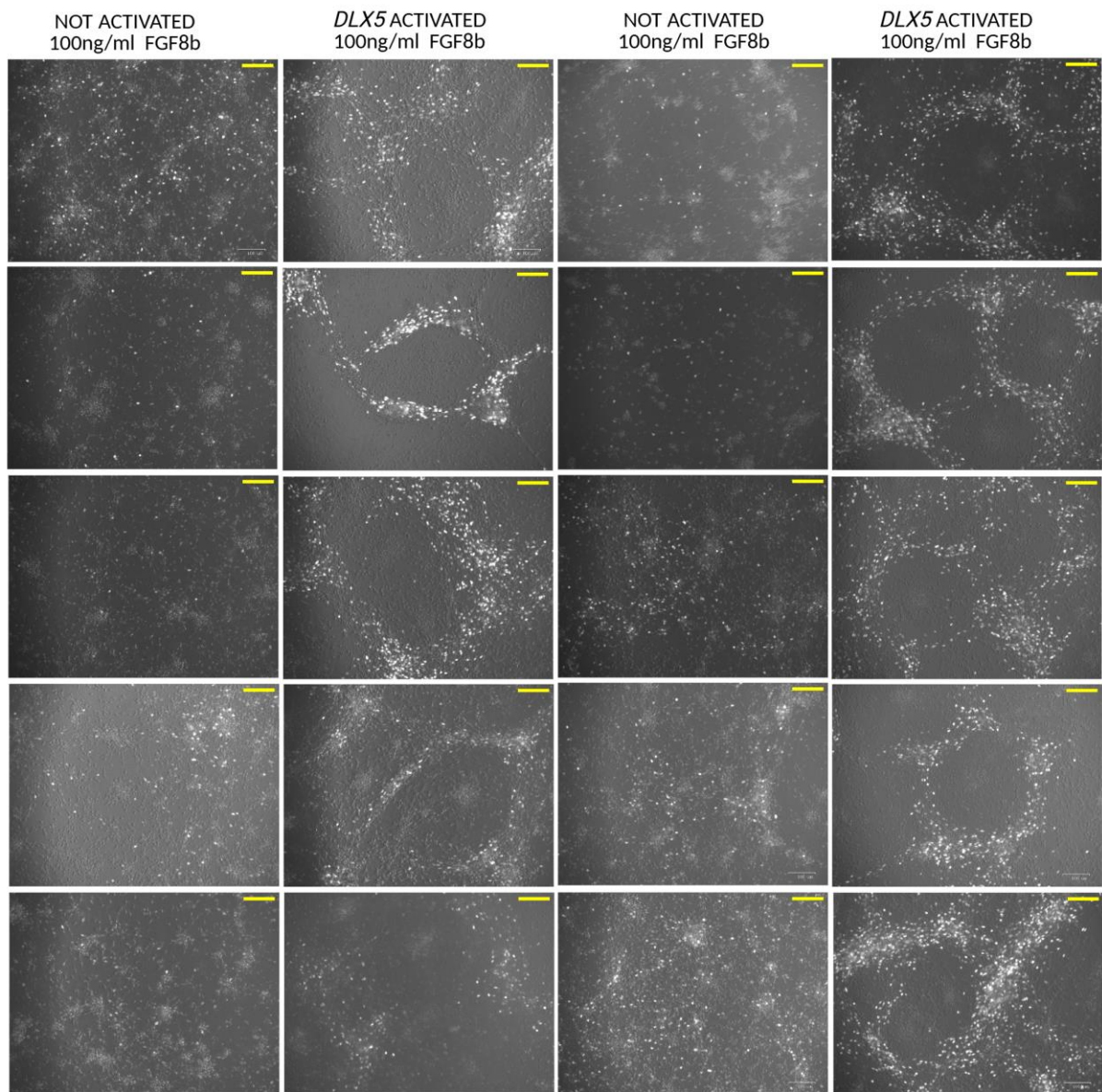

Supplement: Supplementary file 4 [file JME-26-0040_supplementary_figure_4.pdf]
