# Supplementary material for: CRISPR activation of DLX5 drives neural progenitors to the GnRH cell fate
Source: J Mol Endocrinol. 2026 Jul 23;77(1):e260040. doi: 10.1530/JME-26-0040 (PMC13400967; doi:10.1530/JME-26-0040)
Supplement: Supplementary file 5 [file JME-26-0040_supplementary_tables.pdf]

Supplementary table 1: List of RT-PCR primers used

Gene Name	Forward(5'-3')	Reverse(5'-3')
DLX5	CGCTAGCTCCTACCACCAGT	TTTGCCATTCACCATTCTCA
PPIG	ACTCCCAGCCTGCTTCATAC	TACGTCTGAAACGATCCCTTG
GNRH1	TGCCCAGTTTCCTCTTCAAT	GTCAACTGGCAGAAACCCAA
FGF8	GACACCTTTGGAAGCAGAGT	AGCACAATCTCCGTGAAGAC

Supplementary table 2: List of anitbodies used for ICC

Antibody	Catalog no.	Dilution used	Company
DLX5 Polyclonal antibody	10592-1-AP	1:500	Proteintech
SPARC Polyclonal antibody	15274-1-AP	1:100	Proteintech
Collagen I +Collagen III antibdoy	ab34710	1:100	Abcam
DAPI	62248	1:1000	Thermo Scientific
